# Beyond the Lungs: A Rare Case of Extrapulmonary Tuberculosis Presenting With Neck Vein Thrombosis and Seizure

**DOI:** 10.1155/crdi/4888774

**Published:** 2025-08-13

**Authors:** Hamda Al-Mansoori, M. Z. Sharaf Eldean, Abdelkareem Alhyari, Mahmoud Tabouni

**Affiliations:** ^1^Department of Internal Medicine, Hamad Medical Corporation, Doha, Qatar; ^2^Department of Laboratory Medicine and Pathology, Hamad Medical Corporation, Doha, Qatar

**Keywords:** cerebral tuberculoma, extrapulmonary tuberculosis, hypercoagulability, internal jugular vein thrombosis, lymphadenopathy, mediastinoscopy, *Mycobacterium tuberculosis*, ring-enhancing brain lesions, seizure

## Abstract

**Introduction:** Tuberculosis (TB), caused by *Mycobacterium tuberculosis*, primarily affects the lungs but can involve virtually any organ system, manifesting as extrapulmonary TB. While TB-related hypercoagulability and venous thromboembolism are recognized, such presentations remain uncommon and diagnostically challenging, especially in the absence of classical symptoms.

**Case Presentation:** We report the case of a 24-year-old immunocompetent female who initially presented with painless right-sided neck swelling. Imaging revealed an acute thrombus in the right internal jugular vein (IJV), with no clear underlying cause. Further evaluation showed enlarged necrotic mediastinal lymph nodes, raising suspicion for lymphoma. However, the patient later developed a seizure episode, and subsequent neuroimaging revealed multiple intracranial ring-enhancing lesions. Ultimately, mediastinoscopic lymph node biopsy confirmed necrotizing granulomatous inflammation, with a positive TB polymerase chain reaction (PCR), consistent with disseminated TB involving both vascular and central nervous systems. The patient was started on antitubercular therapy, anticoagulation, and adjunctive corticosteroids, with multidisciplinary follow-up arranged.

**Discussion:** This case highlights TB-induced hypercoagulability as a potential cause of isolated venous thrombosis and underscores the diagnostic challenges when TB mimics malignancy. It also emphasizes the importance of considering TB in the differential diagnosis of unexplained thrombosis and intracranial lesions, even in the absence of pulmonary symptoms.

**Conclusion:** Clinicians should maintain a high index of suspicion for extrapulmonary TB in atypical thrombotic events. Early tissue diagnosis and a multidisciplinary approach are key to effective management and favorable outcomes.

## 1. Introduction

Tuberculosis (TB) is an infectious disease caused by *Mycobacterium tuberculosis*. It is estimated that one-third of the global population is infected with this pathogen [1]. While the lungs are most commonly affected, TB can involve virtually any organ system, including the gastrointestinal and genitourinary tracts, lymph nodes (with cervical lymph nodes being the most affected), bones, muscles, and the central nervous system—collectively referred to as extrapulmonary TB [2, 3]. Additionally, TB is known to induce a hypercoagulable state, which can lead to thromboembolic complications. Thromboembolism has been reported in approximately 0.6%–1.0% of patients with TB [4]. However, to the best of our knowledge, very few cases of thromboembolism in TB patients have been documented in the literature [5–8]. Notably, none of these reported cases presented initially with deep venous thrombosis (DVT), nor were they associated with DVT in unusual anatomical locations, except for a single case published in 2011 in the *Indian Journal of Tuberculosi*s [9].

## 2. Case Presentation

A 24-year-old female with no prior medical history presented to the emergency department with a complaint of painless right-sided neck swelling that had been present for three days. She denied any associated symptoms such as fever, dysphagia, or voice changes. There was no history of recent illnesses, cough, shortness of breath, weight loss, or constitutional symptoms. The patient denied recent travel or any known contact with ill people, including those with TB.

Given the persistence of the swelling, she sought further evaluation. On physical examination, she appeared well-nourished, and a localized swelling was noted on the right side of the neck, but no clearly enlarged lymph nodes were appreciated. There was no associated swelling elsewhere. Breast, respiratory, and other systems' examination was unremarkable.

**Figure 1:**
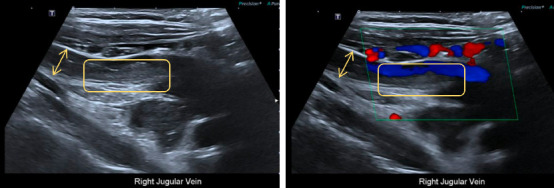
Neck US: the right internal jugular vein (yellow arrow) is grossly distended secondary to acute thrombus (yellow rectangular), with no evidence of extraluminal compression.

An initial ultrasound (US) of the neck (Figure 1) was performed, which revealed a thrombus involving the right internal jugular vein (IJV).

Further laboratory workup, including a complete blood count and an extensive prothrombotic panel—comprising lupus anticoagulant, factor V Leiden mutation, protein C, protein S, antithrombin III, and homocysteine levels—revealed all results within normal limits. Based on these findings, the treating team decided to initiate anticoagulation with rivaroxaban which was initiated due to its convenient once-daily dosing (after initial period), with a planned treatment duration of at least 3 months. The patient was referred to both the anticoagulation and general medicine clinics for outpatient follow-up to monitor for potential bleeding, assess for any new symptoms, and to perform a follow-up US.

**Figure 2:**
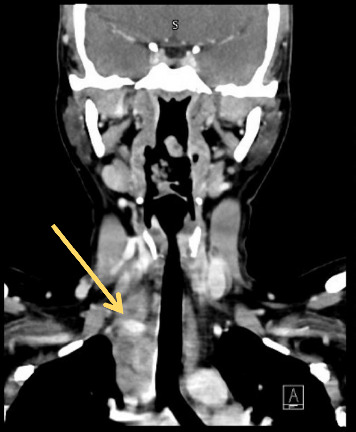
Neck CT with contrast: conglomerate of enlarged necrotic lymph nodes seen in the right paratracheal region/superior mediastinum (yellow arrow), with no evidence of compression on the right IJV.

Subsequently, in the outpatient setting, a contrast-enhanced CT scan of the neck (Figure 2) was performed. The imaging revealed enlarged, necrotic lymph nodes located in the right paratracheal region extending into the superior mediastinum, raising concern for a high-grade lymphoma. As a result, the patient was scheduled for a mediastinoscopy lymph node biopsy under the thoracic surgery team. However, due to urgent social circumstances, she was unable to attend the procedure and had to travel back to her home country.

While the patient was abroad, she experienced a concerning neurological event suggestive of a seizure. The episode was characterized by a sudden loss of consciousness, tongue biting, mouth frothing, and postictal amnesia lasting approximately 10 min. In response to this episode, she was empirically started on levetiracetam 500 mg twice daily.

Four months later, the patient presented to the emergency department shortly after returning from her home country, aiming to expedite the previously planned lymph node excision. Aside from this request, she was asymptomatic at the time of the presentation. She reported strict compliance with both rivaroxaban and levetiracetam during her time abroad. Her vital signs and physical examination were unremarkable. She denied recent infections, fevers, or headaches but did report increased shortness of breath with minimal exertion.

Laboratory investigations were notable for a raised C-reactive protein (CRP) level of 19.4 mg/L and mildly elevated liver enzymes including alkaline phosphatase at 124 U/L, alanine aminotransferase (ALT) at 44 U/L, and aspartate aminotransferase (AST) at 32 U/L. Notably, the Quantiferon-TB Gold Plus test returned positive. Neck US was repeated, and it showed thrombosed proximal to mid right jugular vein with partial flow.

**Figure 3:**
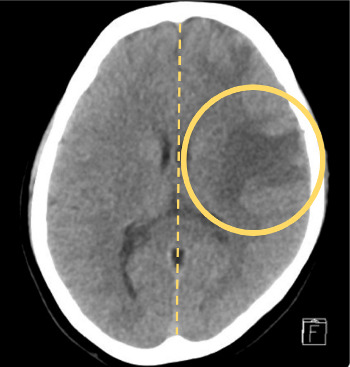
CT head: large hypodensity of the left frontoparietotemporal cerebral hemisphere (yellow circle). There is an associated mild, less than 5 mm, midline shift noted toward the right side (dashed yellow line).

Initial neuroimaging with a noncontrast CT of the head (Figure 3) revealed a large area of hypodensity involving the white matter of the left cerebral hemisphere, primarily in the frontoparietotemporal lobes, associated with mass effect on adjacent sulci.

**Figure 4:**
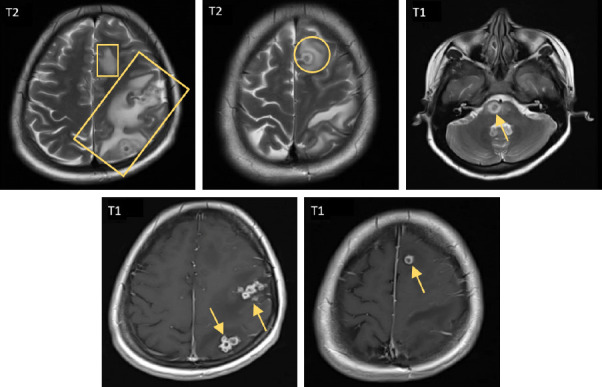
MRI head with contrast: numerous clusters of ring-enhancing lesions are noted in the left frontoparietal lobes and the right side of the pons (yellow arrows) on T1-weighted postcontrast images. The largest lesion, measuring 8 mm in diameter, demonstrates target sign with central low signal intensity and surrounding vasogenic edema (yellow circle) on T2-weighted image. Area of vasogenic edema is also seen in the left parietal region related to other similar lesions (yellow rectangles) on T2-weighted image. No definite abnormal meningeal enhancement is seen.

Following the initial imaging findings, a contrast-enhanced MRI of the brain (Figure 4) was performed for better characterization of the abnormal results. The MRI revealed multiple clusters of complete ring-enhancing lesions, predominantly located in the subcortical regions. These lesions have significant mass effect on the surrounding brain tissue.

**Figure 5:**
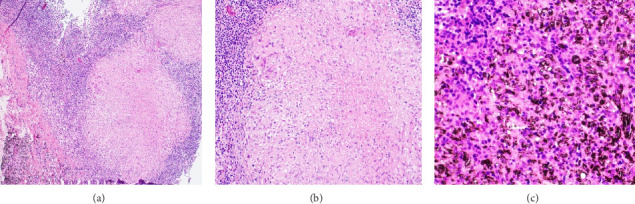
(a) and (b) Representative hematoxylin and eosin (H&E) stained sections showing necrotizing granulomatous inflammation. The granulomas display central necrosis surrounded by epithelioid histiocytes and Langhans-type multinucleated giant cells ((a) H&E, 10 ×; (b) H&E, 20 ×). (c) Anthracotic pigmentation is seen within the lymph node parenchyma ((c) H&E, 40 ×). Summary: (1) Necrotizing granulomatous lymphadenitis. (2) Anthracotic pigment. (3) TB PCR is reported “positive”.

Given the differential diagnosis based on the imaging findings, the two most likely possibilities were lymphoma versus tuberculoma. Both the infectious disease and oncology teams were consulted, and it was agreed upon by both specialties to perform a lymph node biopsy for a definitive diagnosis. The general surgery and interventional radiology teams were also consulted; however, they determined that a biopsy could not be performed due to the critical location of the lymph node and its unpalpable nature. As a result, the thoracic surgery team was involved, and a successful mediastinoscopy biopsy of the right paratracheal lymph node was carried out. Histopathological examination of the biopsy specimen (Figure 5) confirmed the diagnosis of TB.

Under the guidance of the infectious disease team, the patient was initiated on a standard antitubercular regimen comprising moxifloxacin, rifampin, isoniazid, pyrazinamide, and pyridoxine. Moxifloxacin was used in place of ethambutol in the initial antitubercular regimen due to its superior central nervous system penetration and enhanced bactericidal activity, given the presence of multiple brain lesions suggestive of tuberculomas. Ethambutol was avoided to reduce the risk of optic and neurologic toxicity, which could complicate the clinical picture in a patient with possible CNS TB. Dexamethasone was started at a dose of 0.4 mg/kg/day for 1 week, followed by a tapering schedule. She remained on her preexisting medications, rivaroxaban and levetiracetam. The patient was planned to continue antitubercular therapy for a total duration of 12 months, given the central nervous system involvement. Anticoagulation with rivaroxaban and antiepileptic therapy with levetiracetam were also continued concurrently, with the intention to maintain both until completion of the anti-TB treatment. Follow-up was arranged with infectious disease, internal medicine, neurology, and anticoagulation clinics. She was regularly seen in these clinics, demonstrating good clinical progress over a six-month period. She tolerated all medications well, without any reported adverse effects, and remained neurologically stable. She subsequently returned to her home country to continue care there.

## 3. Discussion

This case illustrates an uncommon presentation of extrapulmonary TB manifesting with IJV thrombosis and intracranial tuberculomas in an immunocompetent young adult. The diagnostic complexity, compounded by overlapping radiologic features with lymphoma, underlines the necessity for a broad differential diagnosis and multidisciplinary approach in atypical venous thrombotic events.

Hypercoagulability in TB is a well-established but often underrecognized phenomenon [4, 8]. *Mycobacterium tuberculosis* can induce a prothrombotic state through several mechanisms, including systemic inflammation, endothelial injury, and increased production of procoagulant proteins such as fibrinogen, alongside decreased natural anticoagulants [8]. In this patient, the presence of right IJV thrombosis without any traditional risk factors or underlying thrombophilia supports TB-related hypercoagulability as a plausible etiology. This is consistent with literature showing venous thrombosis occurring in TB [8, 10, 11].

Kutiyal et al. demonstrated significant hematologic abnormalities in TB patients, including elevated fibrinogen levels and shortened activated partial thromboplastin time, indicating a hypercoagulable state, and these hematologic shifts can predispose individuals to thromboembolic complications, even before pulmonary or systemic manifestations of TB become clinically evident [12]. This may explain why our patient developed IJV thrombosis as an early and isolated finding.

Mitroi et al. further emphasized TB's ability to induce a prothrombotic milieu via immune-mediated endothelial dysfunction, cytokine-induced coagulation activation, and platelet aggregation, and the interplay of these mechanisms in our case likely precipitated the vascular complication despite the absence of inherited or acquired thrombophilia [8].

The progression to neurological involvement, presenting with seizures and ring-enhancing brain lesions, signifies disseminated TB [2, 3]. Intracranial tuberculomas often mimic neoplastic processes like lymphoma on imaging due to their mass effect and ring enhancement, posing significant diagnostic challenges [13, 14]. In this case, CT scan findings and lymphadenopathy raised initial suspicion for malignancy, particularly high-grade lymphoma. However, subsequent biopsy confirmed necrotizing granulomatous lymphadenitis with positive TB polymerase chain reaction (PCR), validating the infectious etiology.

Huang et al. described similar cases where pulmonary TB was complicated by silent but severe thromboembolic events such as pulmonary embolism, highlighting the insidious nature of TB-related vascular complications; these complications may present independently or concurrently with more classic symptoms, as seen in our patient who remained largely asymptomatic systemically [14].

The diagnostic journey in this case also emphasizes the utility of mediastinoscopy in obtaining tissue diagnosis from deep lymphatic stations [15]. Ha et al. noted that neurological involvement in TB can be associated with systemic dissemination and emphasized the importance of integrating neuroimaging, systemic evaluation, and tissue biopsy for definitive diagnosis [13].

Management of TB-associated venous thrombosis necessitates an integrated approach targeting both the infection and the thrombotic event, and our patient was appropriately treated with a full course of anticoagulation and standard anti-TB therapy as discussed by Ageno et al. and Gomes and Solomon who noted that although direct oral anticoagulants like rivaroxaban are commonly used in managing thrombosis, their use in TB must be cautiously monitored due to potential drug–drug interactions, especially with rifampin, a known enzyme inducer that can reduce anticoagulant efficacy [11, 16].

The use of adjunctive corticosteroids, such as dexamethasone in this case, is recommended in cases of cerebral TB to reduce inflammatory mass effect and improve outcomes, particularly when intracranial lesions are present with significant edema or neurologic symptoms. However, their use should be approached with caution, as corticosteroids may contribute to an increased risk of thromboembolic complications [13].

In comparison with the case reported by Gowrinath et al., while both cases highlight the rare but serious complication of IJV thrombosis in cervical tuberculous lymphadenopathy, our case is distinguished by its progression to central nervous system involvement [9]. These additional complications necessitated a more extensive diagnostic workup and a more complex therapeutic approach.

In conclusion, this case underscores the diverse and sometimes deceptive presentations of TB, which can mimic malignancy and present with thrombotic complications. Clinicians should maintain a high index of suspicion for TB in young patients presenting with unusual thromboses or lymphadenopathy, even in the absence of classical symptoms. Early tissue diagnosis, coordinated multidisciplinary care, and tailored treatment are essential for optimal outcomes.
